# Experimental Studies on Model Reference Adaptive Control with Integral Action Employing a Rotary Encoder and Tachometer Sensors

**DOI:** 10.3390/s130404742

**Published:** 2013-04-10

**Authors:** Guo-Qiang Wu, Shu-Nan Wu, Yu-Guang Bai, Lei Liu

**Affiliations:** 1 State Key Laboratory of Structural Analysis for Industrial Equipment, Faculty of Vehicle Engineering and Mechanics, Dalian University of Technology, Dalian 116024, China; E-Mail: gqwu@dlut.edu.cn; 2 School of Aeronautics and Astronautics, Faculty of Vehicle Engineering and Mechanics, Dalian University of Technology, Dalian 116024, China; E-Mails: wushunan@dlut.edu.cn (S.-N.W.); liulei@dlut.edu.cn (L.L.)

**Keywords:** MRAC, integral action, Lyapunov stability, asymptotical convergence

## Abstract

In this paper, an adaptive law with an integral action is designed and implemented on a DC motor by employing a rotary encoder and tachometer sensors. The stability is proved by using the Lyapunov function. The tracking errors asymptotically converge to zero according to the Barbalat lemma. The tracking performance is specified by a reference model, the convergence rate of Lyapunov function is specified by the matrix *Q* and the control action and the state weighting are restricted by the matrix Γ. The experimental results demonstrate the effectiveness of the proposed control. The maximum errors of the position and velocity with the integral action are reduced from 0.4 V and 1.5 V to 0.2 V and 0.4 V, respectively. The adaptive control with the integral action gives satisfactory performance, even when it suffers from input disturbance.

## 1. Introduction

Various electromechanical motors have been used for industrial applications, e.g., electric motors, piezomotors and hydraulic actuators. Generally, it is necessary to enhance the motor performance with the feedback control based on state measurements. Khorrami *et al.*, used linear motors to establish ultra-accurate high-speed six degree-of-freedom manipulation [1]. To measure the angle and speed of motor shafts, rotary encoders and tachometer sensors have been widely used because of their high performance and reliability [2–4].

As digital angle-measuring sensors, rotary encoders consist of optics, mechanics and electronics. Compared to analog angle-measuring sensors, digital rotary encoders have simple structures while preserving high accuracy. In order to achieve accurate motion control, the velocity can be measured by using tachometer sensors which usually consist of tachogenerators and circuits. The tachogenerator can also give the voltage output which is proportional to the speed of the rotational motor.

To achieve precise motion, it is necessary to measure and input both the angle and the speed of motor shafts by employing a rotary encoder and tachometer sensors. Based on the angle and speed sensors, various controllers have been designed for motors [5,6]. Generally, motors can be controlled by conventional PID controllers. Nikulin and Frantsuzova presented a modified PD controller to ensure the desired system speed and damping vibrations [7]. Chaiya and Kaitwanidvilai provided a robust PID controller which could control the motor speed [8], but PID controllers have limited performance in the presence of disturbances and uncertainties. Xu and Yang presented a simple and robust speed control scheme for a permanent magnet synchronous motor to enhance the performance robustness [9]. Nouri *et al.*, proposed a model-following adaptive controller for the speed control of a motor drive system [10]. Melkote and Khorrami proposed adaptive control for direct drive brushless DC motors [11]. Moreover, intelligent algorithms have also been investigated [12–15]. Fallahi and Azadi added neural network sliding mode control to enhance the adaptive control of motors [16]. However, intelligent controllers are usually very complex. It is difficult for engineers to design and optimize intelligent controllers, e.g., Xu and Huang [17] designed an iterative learning controller (ILC), but found that the reference signal had to be pre-filtered in order to satisfy the complex initial conditions of ILC. Furthermore, several accurate models were identified at different voltage ranges, and the iterations were implemented offline.

**Figure 1.**
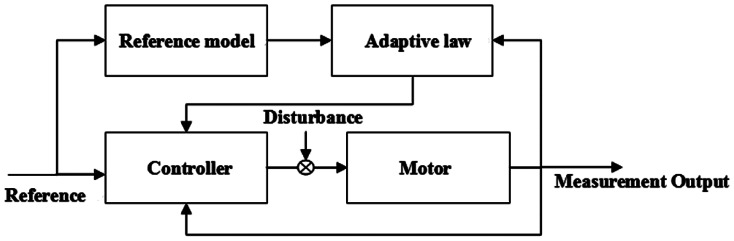
MRAC control sketch of the motor.

This paper uses Model Reference Adaptive Control (MRAC), which is a convenient approach to satisfy the requirements of designers [18]. The general idea of MRAC is to create a closed loop regulator with parameters that could be updated to match a desired response. The desired performance is specified by a stable reference model, and the parameters of the adaptive law are adjusted based on the errors between the reference model and the plant, as shown in Figure 1.

Though the adaptive control has shown its effectiveness in achieving robust performance without the knowledge of parameter values, a comprehensive design approach is still necessary for engineering applications. In order to obtain high-performance adaptive control in the presence of disturbances, this paper presents a comprehensive design approach in which both the bandwidth and damping ratio can be included in the proposed controller.

In this paper, two kinds of MRAC are designed and then used on a motor by employing a rotary encoder and tachometer sensors. The tracking error can converge to zero with the integral action in the presence of input disturbances. The experimental results are presented to investigate the effectiveness of the proposed control approach.

## 2. Adaptive Control Design without the Integral Action

### 2.1. Angle and Speed Sensors

**Figure 2.**
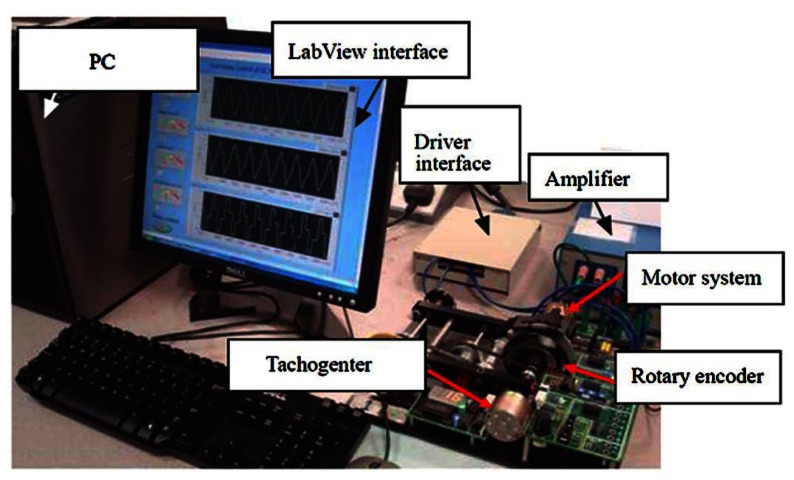
Sketch of the motor experiment.

Figure 2 shows the experimental setup. It consists of a DC motor, a computer with LabView software, a drive interface, an amplifier, a tachometer sensor and a rotary encoder. The tachometer sensor is used to measure the rotation speed of the motor shaft. The output voltage of the tachogenertor (*i.e.*, tachometer generator—a device to measure the rotational rate of the motor shaft with the internally generated electrical signal generated by the motor shaft) is proportional to the motor speed. Then, the voltage is applied to the voltmeter in which the dial can be calibrated in speed units (*i.e.*, usually in revolutions per minute (rpm)). In addition, a rotary encoder is used to measure the linear rotation angle of the motor draft. The rotary encoder can convert the ration angle to digital voltage. Thus, both the angle and speed can be measured and controlled.

**Figure 3.**
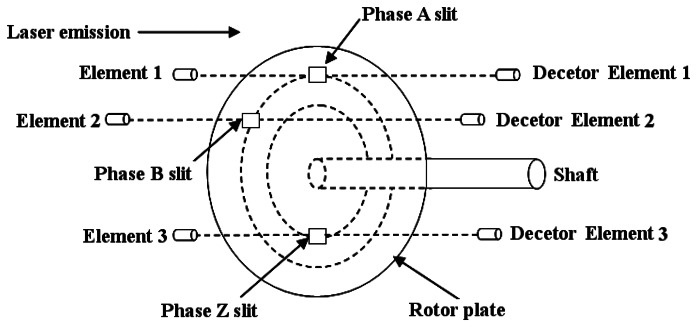
Working principle of the rotary encoder.

After calibration, the generated voltage is a factor of the motor speed. Then, the motor speed can be fed to the adaptive controller. Next, Figure 3 is given to present the working principle of the rotary encoder in which phase A, phase B, and phase Z are the three-phase models to represent the output signal counts for an incremental encoder [19]. The rotary encoder consists of the main grating, index grating, illuminant and photosensitive device. In this paper, an incremental encoder is used because of its simplification, small size and high-speed response. There is one impulse corresponding to every grating. The summation of impulses represents the angle position of the motor.

### 2.2. Adaptive Controller Design and Stability Proof

This section presents a design method of the adaptive control without the integral action. The stability proof is also presented. To achieve good tracking performance, a MRAC is designed to drive the tracking error to zero. Considering simplification, the transfer function in Equation (1) is used to give the motor dynamics:

(1) $$G_{p}=\frac{K}{s(1+\tau s)}$$

where *K* denotes the DC gain of the velocity transfer function, and *τ* denotes the time constant. *τ* are unknown parameters, but their signs can be tested in the experiment. The state space model of Equation (1) can be rewritten in Equation (2)

(2) $$\begin{array}{l} \dot{x}=A_{p}x+\text{gbu}\\ y=Cx \end{array}$$

where:

$$A_{p}=\left[\begin{array}{cc} 0 & 1\\ 0 & \frac{1}{\tau } \end{array}\right],\text{b}=\left[\begin{array}{c} 0\\ 1 \end{array}\right],\text{g}=K,C=\left[\begin{array}{cc} 1 & 0\\ 0 & 1 \end{array}\right]$$

To specify the desired performance, this paper employs a stable reference model as shown in Equation (3), from which the domain and frequency index can be specified:

(3) $$G_{m}=\frac{\omega_{n}^{2}}{s^{2}+2\xi \omega_{n}s+\omega_{n}^{2}}$$

where *ξ* is the damping ratio and *ω_n_* is the natural frequency of the reference model. The corresponding state space description can be given as:

(4) $$\begin{array}{c} \left(\begin{array}{c} {\dot{x}}_{1}\\ {\dot{x}}_{2} \end{array}\right)=\left[\begin{array}{cc} 0 & 1\\ -\omega_{n}^{2} & -2\xi \omega_{\text{n}} \end{array}\right]\left(\begin{array}{c} x_{1}\\ x_{2} \end{array}\right)+\omega_{n}^{2}\left[\begin{array}{c} 0\\ 1 \end{array}\right]r\\ y=\left[\begin{array}{cc} 1 & 0\\ 0 & 1 \end{array}\right]\left(\begin{array}{c} x_{1}\\ x_{2} \end{array}\right) \end{array}$$

Equation (4) can be further written as:

$${\dot{x}}_{m}=A_{m}x_{m}+g_{m}br$$

The following non-adaptive control law is used:

(5) $$u=\theta_{x}^{*T}x_{p}+\theta_{r}^{*}r$$

where $\theta_{x}^{*}$ and $\theta_{r}^{*}$ are the exact gains of the controller Equation (5).

Then, the closed loop system can be given by:

(6) $${\dot{x}}_{p}=\left[A_{p}+gb\theta_{x}^{*T}\right]x_{p}+(g\theta_{r}^{*})br$$

The feedback control system achieves the performance as the matching condition below:

(7) $$\begin{array}{l} A_{p}+gb\theta_{x}^{*T}\equiv A_{m}\\ g\theta_{r}^{*}\equiv g_{m} \end{array}$$

The exact control gains $\theta_{x}^{*T}$ and $\theta_{r}^{*}$ guarantee that the closed loop system matches the reference model. Actually, the exact values of $\theta_{x}^{*T}$ and $\theta_{r}^{*}$ are unknown. The controller in Equation (5) can be rewritten to:

(8) $$u=\theta_{x}^{T}(x)x_{p}+\theta_{r}r(t)$$

where $\theta_{x}^{T}$ and *θ_r_* can be determined by the adaptation law in Equation (9):

(9) $$\dot{\theta }={\dot{\theta }}^{*}+\dot{\phi }=2\dot{\phi }=-2\mathrm{sgn}(g)\Gamma xe^{T}Pb$$

where θ^*^ is a constant or the slow time-variant parameter (i.e., θ̇^*^=*ϕ̇*).

**Figure 4.**
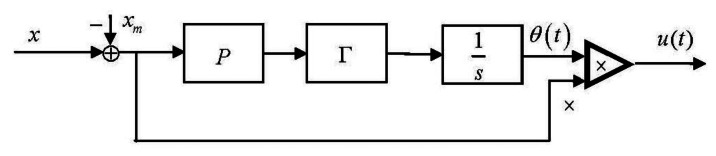
Adaptive control block.

The adaptive control law is nonlinear, as shown in Figure 4.

To give the tracking performance, the parameter errors *ϕ_x_* and *ϕ_r_* are defined by:

(10) $$\begin{array}{c} \phi_{x}=\theta_{x}-\theta_{x}^{*}\\ \phi_{r}=\theta_{r}-\theta_{r}^{*} \end{array}$$

Then, the closed loop system can be rewritten to:

(11) $$\begin{array}{c} {\dot{x}}_{p}=A_{p}x_{p}+gb(\theta_{x}^{T}(x)x_{p}+\theta_{r}r(t))\\ =A_{m}x_{p}+gb\phi_{x}^{T}x_{p}+gb\theta_{r}r(t) \end{array}$$

Next, let *e* be the state error and *e*=*x_p_*−*x_m_*.

By comparing the closed loop system in Equation (11) with the reference model in Equation (5), the dynamics of the tracking error *e* can be given:

(12) $$\begin{array}{c} \dot{e}=A_{m}e+gb\phi_{x}^{T}x_{p}+gb\theta_{r}r(t)\\ =A_{m}e+gb\phi^{T}x \end{array}$$

where:

$$\phi =\left[\begin{array}{c} \phi_{r}\\ \phi_{r} \end{array}\right],x=\left[\begin{array}{c} x_{p}\\ r \end{array}\right]$$

In the reference model in Equation (4), a stable matrix is used for *A_m_* that can satisfy the algebraic Riccati Equation (13) (*i.e.*, for any symmetric positive definite matrix *Q*, there is a symmetric positive definite matrix *P* satisfying Equation (13)):

(13) $${\text{A}}_{m}^{T}P+PA_{m}=-Q$$

To prove the stability, a Lyapunov function candidate is used as follows:

(14) $$V(e,\phi )=e^{T}Pe+\left|g\right|\phi^{T}\Gamma^{-1}\phi$$

where *Γ* is a positive definite matrix.

The time derivative of the Lyapunov function candidate can be given:

(15) $$\begin{array}{ll} \dot{V}(e,\phi ) & =2e^{T}P\dot{e}+2g\phi^{T}\Gamma^{-1}\dot{\phi }\\ & =e^{T}({\text{A}}_{m}^{T}P+PA_{m})e+2ge^{T}Pb\phi^{T}+2g\phi^{T}\Gamma^{-1}\dot{\phi }\\ & =-e^{T}Qe+2ge^{T}Pb\phi^{T}+2g\phi^{T}\Gamma^{-1}\dot{\phi } \end{array}$$

Then, Equation (15) can be rewritten to:

(16) $$\begin{array}{ll} \dot{V}(e,\phi ) & =-e^{T}Qe+2ge^{T}Pb\phi^{T}x-2\mathrm{sgn}(g)\left|g\right|\phi^{T}xe^{T}Pb\\ & =-e^{T}Qe\leq 0 \end{array}$$

where *g* = sgn(|*g*|)|*g*|, *ϕ^T^x* is a scalar.

Thus, the system is stable according to the Lyapunov theorem [20]. Moreover, ‖*e*‖, *ė*, ‖*ϕ*‖, *x_n_,θ_x_* and *θ_r_* are bounded, and:

$$\int_{0}^{\infty }e^{T}\text{Qedt}=V_{0}-V_{\infty }<V_{0}\leq c_{1}$$

According to the Barbalat lemma [20] here *V̇* [0,+∞] →*R* is a uniformly continuous function on [0,+∞]. Supposing $\underset{t\to \infty }{\lim}\int_{0}^{t}\dot{V}(\tau )d\tau$ exist and be finite. Then, there is $\dot{V}\underset{t\to \infty }{\to }0$.

Here *ė* is bounded. Furthermore:

$$\int_{0}^{\infty }e^{T}\text{Qedt}=V_{0}-V_{\infty }<V_{0}\leq c_{1}$$

According to the Barbalat lemma, the tracking error *e* is asymptotically stable and lim *e* = 0(*i.e.*, lim(‖*x_m_*−*x_n_‖*) = 0).

To improve the adaptation rate, the proportional action can be added to the adaptive law [14]. Finally, the adaptive law can be given in Equation (17):

(17) $$\bar{\theta }=-(\gamma_{1}\int \mathrm{sgn}(k_{p})\Gamma \bar{\omega }e_{1}dt+\gamma_{2}\mathrm{sgn}(k_{p})\Gamma \bar{\omega }e_{1}$$

where *γ*_1_ = 1 and *γ*_2_ = 0.5, and they are used in experiments.

### 2.3. Experimental Studies of Adaptive Control without the Integral Action

Experimental studies of the adaptive control are now presented. Firstly, *ρ* can be used to give the convergence rate of the adaptive law, as shown in Equation (18):

(18) $$\rho =\frac{\left|\dot{V}\right|}{V}=\frac{{\bar{e}}^{T}\bar{Q}\bar{e}}{{\bar{e}}^{T}\bar{P}\bar{e}+\left|g\right|{\bar{\phi }}^{T}\Gamma^{-1}\bar{\phi }}\leq \frac{{\bar{e}}^{T}\bar{Q}\bar{e}}{{\bar{e}}^{T}\bar{P}\bar{e}}\leq \frac{\lambda_{\max}(\bar{Q})}{\lambda_{\min}(\bar{P})}=\rho_{\max}$$

After several trials, the reference model is given:

$$A_{m}=\left[\begin{array}{cc} 0 & 1\\ -25 & -9 \end{array}\right],\omega =5,\xi =0.9$$

and the matrix *Q* is selected as:

$$Q=\left[\begin{array}{ll} 10 & 0\\ 0 & 1 \end{array}\right]$$

The selection of *Q* means that the position tracking is more important than the velocity tracking. Moreover, the sampling interval *h* should be less than the max interval in order to handle the fastest adaptation rate [18]:

(19) $$h<\frac{1}{20}\frac{1}{\rho_{\max}}=\frac{1}{20}\frac{\lambda_{\min}(\bar{P})}{\lambda_{\max}(\bar{Q})}$$

The matrix *P* is solved by the ARE in Equation (13):

$$P=\left[\begin{array}{ll} 3.74 & 0.2\\ 0.2 & 0.078 \end{array}\right]$$

and denoting *Γ* :

$$\Gamma =\left[\begin{array}{ccc} 1 & 0 & 0\\ 0 & 0.5 & 0\\ 0 & 0 & 1 \end{array}\right]$$

**Figure 5.**
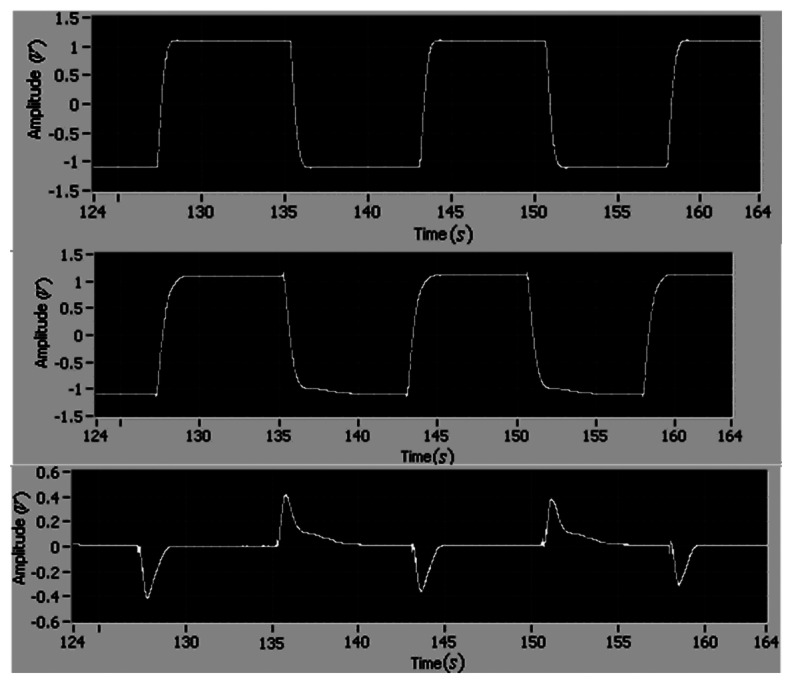
The reference position, the motor position and the tracking error (*i.e.*, (**top**), **(middle)** and **(bottom**), respectively).

**Figure 6.**
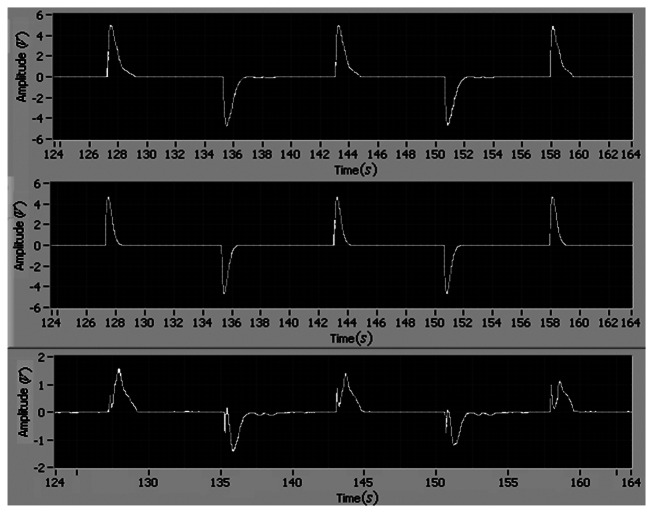
The reference velocity, the motor velocity and the tracking error (*i.e.*, (**top**), (**middle**) and (**bottom**), respectively).

The tracking performance of the adaptive control is shown in Figures 5 and 6. It can be found that the tracking errors of angular position and velocity asymptotically converge to zero in one period, and the maximum tracking errors of position and velocity are 0.4 and 1.5, respectively.

**Figure 7.**
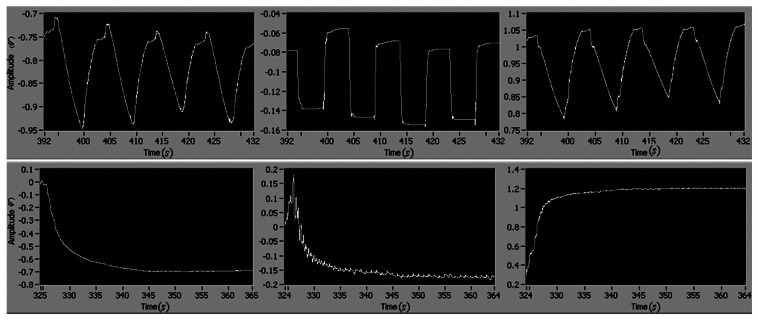
*θ̅* responses under square wave tracking (**top**) and sinusoidal tracking (**bottom**), respectively.

To guarantee the stability of the adaptive control, it is necessary to demonstrate the boundness of *θ̅*. Figure 7 shows the responses of *θ̅*. It can be seen that the estimation of *θ̅* = [*θ*_x1_*θ*_x2_*θ_I_*] is bounded.

### 2.4. Investigations of Q and Γ

*Q* and *Γ* are two important parameters of the adaptive controller. Different values of *Q* and *Γ* are adopted to investigate their influences, here two group values of them are:

$$\begin{array}{c} Q_{1}=\left[\begin{array}{ll} 10 & 0\\ 0 & 1 \end{array}\right],P_{1}=\left[\begin{array}{ll} 3.74 & 0.2\\ 0.2 & 0.078 \end{array}\right]\\ Q_{1}=\left[\begin{array}{ll} 30 & 0\\ 0 & 30 \end{array}\right],P_{1}=\left[\begin{array}{ll} 48.7 & 0.6\\ 0.7 & 1.778 \end{array}\right]\\ \Gamma_{1}=\left[\begin{array}{ccc} 1 & 0 & 0\\ 0 & 0.5 & 0\\ 0 & 0 & 1 \end{array}\right],\Gamma_{2}=\left[\begin{array}{ccc} 10 & 0 & 0\\ 0 & 5 & 0\\ 0 & 0 & 10 \end{array}\right] \end{array}$$

where *Q*_2_ is larger than *Q*_1_.

The adaptation rates can be respectively estimated:

$$\begin{array}{c} \rho_{1}=\frac{\lambda_{\max}(\bar{Q})}{\lambda_{\min}(\bar{P})}=\frac{10}{0.0669}=149.5\\ \rho_{2}=\frac{\lambda_{\max}(\bar{Q})}{\lambda_{\min}(\bar{P})}=\frac{30}{1.77}=16.9 \end{array}$$

where *ρ* is the adaptive rate, and *ρ*_1_ is faster than *ρ*_2_.

**Figure 8.**
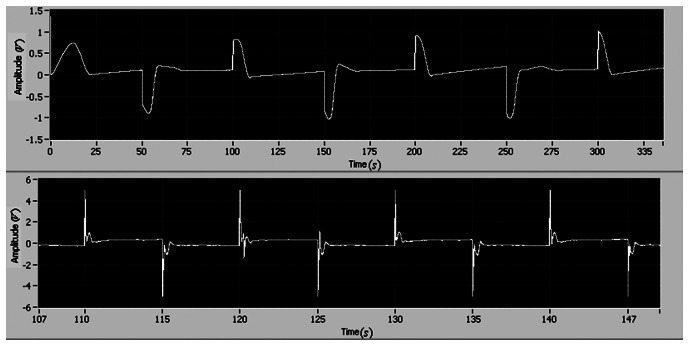
Control signals under (*Q_1_, Γ_1_*) and (*Q_2_, Γ_2_*) ((**top**) and (**bottom**), respectively).

During experiments, the adaptive control using *Q*_1_ and *Q*_2_ have similar tracking errors, but the control signals are different. Figure 8 shows the voltages of the adaptive control. The control voltage of the adaptive control with (*Q_2_, Γ_2_*) is four time larger than that of the adaptive control with (*Q_1_, Γ_1_*), though there is no significant differences in the tracking errors.

## 3. Adaptive Control with the Integral Action

### 3.1. Adaptive Controller Design with the Integral Action and Stability Validation

To further investigate the adaptive control in the presence of disturbances, this section presents the adaptive control with integral action. The integral control action can be added to the adaptive law through Equation (20):

(20) $${\dot{x}}_{I}=x_{1}-r$$

where *r* is the reference signal and *x_I_* is a new state:

(21) $$x_{I}=\int_{0}^{t}x_{1}-rd\tau$$

The augmented plant can be rewritten to:

(22) $$\left(\begin{array}{c} {\dot{x}}_{1}\\ {\dot{x}}_{2}\\ {\dot{x}}_{3} \end{array}\right)=\left[\begin{array}{ccc} 0 & 1 & 0\\ 0 & \frac{1}{\tau } & 0\\ 1 & 0 & 0 \end{array}\right]\left(\begin{array}{c} x_{1}\\ x_{2}\\ x_{3} \end{array}\right)+g\left[\begin{array}{l} 0\\ 1\\ 0 \end{array}\right]u+\left[\begin{array}{l} 0\\ 0\\ -1 \end{array}\right]r$$

Similar to Section 2.2, the control law can be given by:

(23) $$u=\theta_{x}^{*T}x_{p}+\theta_{I}^{*}x_{3}$$

Then the closed loop system can be given as:

(24) $$\left(\begin{array}{c} {\dot{x}}_{1}\\ {\dot{x}}_{2}\\ {\dot{x}}_{3} \end{array}\right)=\left[\begin{array}{ccc} 0 & 1 & 0\\ a_{21}^{p} & a_{22}^{p} & a_{23}^{p}\\ 1 & 0 & 0 \end{array}\right]\left(\begin{array}{c} x_{1}\\ x_{2}\\ x_{3} \end{array}\right)+\left[\begin{array}{l} 0\\ 0\\ -1 \end{array}\right]r$$

where:

(25) $$\left[\begin{array}{ccc} 0 & 1 & 0\\ a_{21}^{p} & a_{22}^{p} & a_{23}^{p}\\ 1 & 0 & 0 \end{array}\right]=\left[\begin{array}{cc} A_{p}+gb\theta_{x}^{*T} & gb\theta_{I}^{*}\\ 1 & 0 \end{array}\right]$$

According to the matching condition in Equation (7), the following equation can be given:

(26) $$\left[\begin{array}{cc} A_{p}+gb\theta_{x}^{*T} & gb\theta_{I}^{*}\\ 1 & 0 \end{array}\right]={\bar{A}}_{m}$$

The reference model can be given:

(27) $${\dot{x}}_{m}={\bar{A}}_{m}x_{m}+\left[\begin{array}{c} 0\\ -1 \end{array}\right]r$$

Actually, the exact value of $\theta_{x}^{*}$ and $\theta_{I}^{*}$ cannot be known, so the corresponding estimated values of *θ_x_* and *θ_I_* are used instead. We note that *ϕ_x_* and *ϕ_I_* are the estimate errors as shown in Equation (28):

(28) $$\begin{array}{c} \phi_{x}=\theta_{x}(t)-\theta_{x}^{*}\\ \phi_{I}=\theta_{I}(t)-\theta_{I}^{*} \end{array}$$

and

$${\bar{x}}_{p}=\left(\begin{array}{c} x_{1}\\ x_{2}\\ x_{3} \end{array}\right),\bar{\varphi }=\left[\begin{array}{c} \varphi_{x}\\ \varphi_{I} \end{array}\right]\;\text{and}\;\bar{e}={\bar{x}}_{p}-{\bar{x}}_{m}$$

where *θ_x_*(*t*) and $\theta_{I}^{*}(t)$ are the estimated values $\theta_{x}^{*}$ and $\theta_{I}^{*}$, respectively.

Substituting *u* into the plant model in Equation (22), the closed loop can be given:

(29) $$\left(\begin{array}{c} {\dot{x}}_{p}\\ {\dot{x}}_{I} \end{array}\right)=\left[\begin{array}{cc} A_{p}+gb\theta_{x}^{*T} & gb\theta_{I}^{*}\\ 1 & 0 \end{array}\right]\left(\begin{array}{c} x_{p}\\ x_{I} \end{array}\right)+\left[\begin{array}{cc} gb\phi_{x} & gb\phi_{I}\\ 0 & 0 \end{array}\right]\left(\begin{array}{c} x_{p}\\ x_{I} \end{array}\right)+\left[\begin{array}{c} 0\\ -1 \end{array}\right]r$$

Equation (29) can be rewritten to:

(30) $${\bar{x}}_{p}={\bar{A}}_{m}{\bar{x}}_{p}+\left[\begin{array}{cc} gb\phi_{x} & gb\phi_{I}\\ 0 & 0 \end{array}\right]{\bar{x}}_{p}+\left[\begin{array}{c} 0\\ -1 \end{array}\right]r$$

The error equation can be given

(31) $$\bar{e}={\bar{A}}_{m}\bar{e}+g\bar{b}{\bar{\phi }}^{T}{\bar{x}}_{p}$$

Choosing the Lyapunov function as Equation (32)

(32) $$V={\bar{e}}^{T}\bar{P}\bar{e}+\left|g\right|{\bar{\varphi }}^{T}\Gamma^{-1}\bar{\varphi }$$

Then, the time derivative of *V* can be obtained:

(33) $$\frac{dV}{dt}=2{\bar{e}}^{T}\bar{P}\bar{e}+2\left|g\right|{\bar{\varphi }}^{T}\Gamma^{-1}\bar{\varphi }={\bar{e}}^{T}\left({\bar{A}}_{m}^{T}P{\bar{A}}_{m}\right)\bar{e}+2g{\bar{e}}^{T}P\bar{b}{\bar{\phi }}^{T}{\bar{x}}_{p}+2\left|g\right|{\bar{\varphi }}^{T}\Gamma^{-1}\bar{\varphi }$$

Denote that the adaptive law is:

(34) $$\bar{\phi }=\left[\begin{array}{c} {\dot{\theta }}_{x}\left(t\right)\\ {\dot{\theta }}_{I}\left(t\right) \end{array}\right]=-\text{sgn}\left(g\right)\Gamma \left[\begin{array}{c} x_{p}\\ x_{I} \end{array}\right]{\bar{e}}^{T}\bar{P}\bar{b}$$

Then, *P̅* can be gotten from the ARE Equation (35):

(35) $${\bar{A}}_{m}^{T}\bar{P}+P{\bar{A}}_{m}=-\bar{Q}$$

where *Q̅* is a positive definite symmetric matrix, so the following Equation (36) can be obtained:

(36) $$\frac{dV}{dt}=-{\bar{e}}^{T}\bar{Q}\bar{e}\leq 0$$

It can be concluded that the closed loop is Lyapunov stable, and *e̅* and *φ̅* are bounded. As in Section 2.2, the tracking error *e̅* is asymptotically stable.

The adaptive control law is shown as follows:

(37) $$u=\theta_{x}^{T}x_{p}+\theta_{I}x_{I}$$

(38) $$\dot{\theta }=\left[\begin{array}{c} {\dot{\theta }}_{x}\\ {\dot{\theta }}_{I} \end{array}\right]=-\text{sgn}(g)\Gamma \left[\begin{array}{c} x_{p}\\ x_{I} \end{array}\right]\bar{P}\bar{b}$$

where *ϕ̅* ≈ *θ̇*, *P* can be computed through the ARE.

### 3.2. Reference Model Design

This section presents how to specify the requirements through the reference model *G_m_*. Firstly, the third order model in Equation (39) is given to satisfy the matching condition of the adaptive integral control. To specify performance indexes such as the bandwidth and damping ratio, the reference model *G_m_* can be approximated by the second order model *G_m_*_1_:

(39) $$G_{m}=G_{m1}\cdot G_{m2}=\frac{w_{n}^{2}}{s^{2}+2\zeta w_{n}+w_{n}^{2}}\cdot \frac{a_{m}}{s+a_{m}}=\frac{a_{m}w_{n}^{2}}{\left(s+P_{1})(s+{\bar{P}}_{1})(s+a_{m}\right)}$$

where ‖*a_m_*‖≥3‖*p*_1_‖ (i.e., *p*_1_ and *p̅*_1_ are dominant poles which mainly contribute to the dynamic response of the reference model.)

To satisfy the matching conditions, the reference model is:

(40) $$\begin{array}{c} \left(\begin{array}{c} {\dot{x}}_{1}\\ {\dot{x}}_{2}\\ {\dot{x}}_{3} \end{array}\right)=\left[\begin{array}{ccc} 0 & 1 & 0\\ a_{21} & a_{22} & a_{23}\\ 1 & 0 & 0 \end{array}\right]\left(\begin{array}{c} x_{1}\\ x_{2}\\ x_{3} \end{array}\right)+\left[\begin{array}{c} 0\\ 0\\ -1 \end{array}\right]r\\ y=\left[\begin{array}{ccc} 1 & 0 & 0 \end{array}\right]\left(\begin{array}{c} x_{1}\\ x_{2}\\ x_{3} \end{array}\right) \end{array}$$

Both Equations (39) and (40) describe the same reference model. The state space Equation (40) is transformed into the transfer function:

(41) $$G_{m}=D+C{\left(sI-A\right)}^{-1}B=\left[\begin{array}{ccc} 1 & 0 & 0 \end{array}\right]{\left(sI_{3\times 3}-\left[\begin{array}{ccc} 0 & 1 & 0\\ a_{21} & a_{22} & a_{23}\\ 1 & 0 & 0 \end{array}\right]\right)}^{-1}\left[\begin{array}{c} 0\\ 0\\ -1 \end{array}\right]=\frac{-a_{23}}{s^{3}-a_{22}s^{2}-a_{21}s-a_{23}}$$

Compared with the transfer function in Equation (39), it is seen that:

(42) $$\begin{array}{l} a_{21}=-(\omega_{n}^{2}+2\zeta \omega_{n}a_{m})\\ a_{22}=-(2\zeta \omega_{n}+a_{m})\\ a_{23}=-\omega_{n}^{2}a_{m} \end{array}$$

The dynamics of the reference model should match the dynamics (*i.e.*, natural frequency) of the motor system and the sampling capability. After testing several groups of values, ζ = 0.9, *w_n_* = 3 and *a_m_* = 9 are chosen in this paper. Then, there are *a*_21_ = −57.6, *a*_22_ = −14.4 and *a*_23_ = −81. The reference model can be rewritten to:

(43) $$\begin{array}{c} \left(\begin{array}{c} {\dot{x}}_{1}\\ {\dot{x}}_{2}\\ {\dot{x}}_{3} \end{array}\right)=\left[\begin{array}{ccc} 0 & 1 & 0\\ -57.6 & -14.4 & -81\\ 1 & 0 & 0 \end{array}\right]\left(\begin{array}{c} x_{1}\\ x_{2}\\ x_{3} \end{array}\right)+\left[\begin{array}{c} 0\\ 0\\ -1 \end{array}\right]r\\ y=\left[\begin{array}{ccc} 1 & 0 & 0 \end{array}\right]\left(\begin{array}{c} x_{1}\\ x_{2}\\ x_{3} \end{array}\right) \end{array}$$

**Figure 9.**
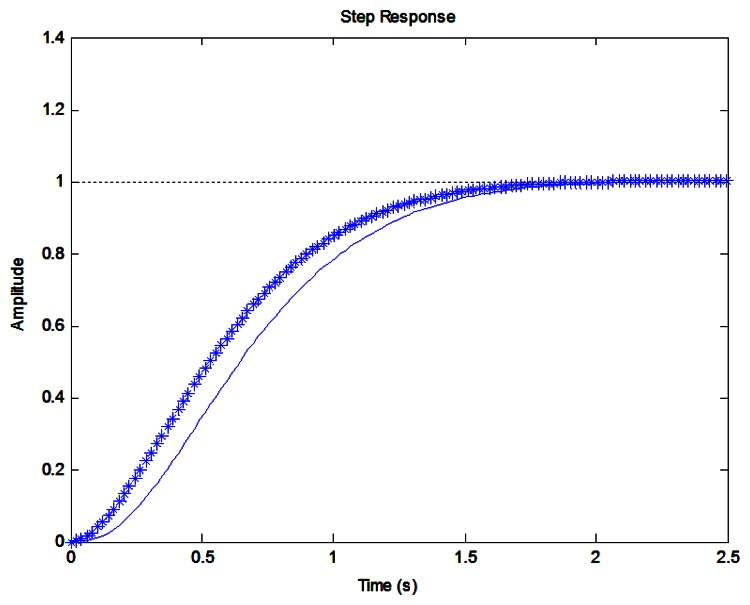
Step responses of *G_m_* (solid line) and *G_m_*_1_ (star line).

The step responses of *G*_*m*1_ and *G_m_* are shown in Figure 9. It is seen that *G*_*m*1_ contributes most of the response for the third order reference model *G_m_*. The fast dynamics of the reference model is influenced by the part *a_m_*/*(s* + *a_m_)*:

### 3.3. Experimental Studies on the Adaptive Control with Integral Action

In Section 2, it has been found that a large *Q̅* results in fast convergence while needing strong action and fast sampling rate. In contrast, a small *Q̅* results in slow convergence and cannot give better performance. A suitable *Q̅* should be given to satisfy the convergence rate and hardware limitation.

After trials, *Q̅* is adopted:

$$\bar{Q}=\left[\begin{array}{ccc} 15 & 0 & 0\\ 0 & 10 & 0\\ 0 & 0 & 15 \end{array}\right]$$

Then, *P̅* can be computed:

$$\bar{P}=\left[\begin{array}{lll} 32.9913 & 0.7111 & 34.4582\\ 0.7111 & 0.3966 & 0.0926\\ 33.4585 & 0.0926 & 62.9307 \end{array}\right]$$

In addition, *Γ* is adopted:

$$\Gamma =\left[\begin{array}{ccc} 4 & 0 & 0\\ 0 & 2.5 & 0\\ 0 & 0 & 4 \end{array}\right]$$

**Figure 10.**
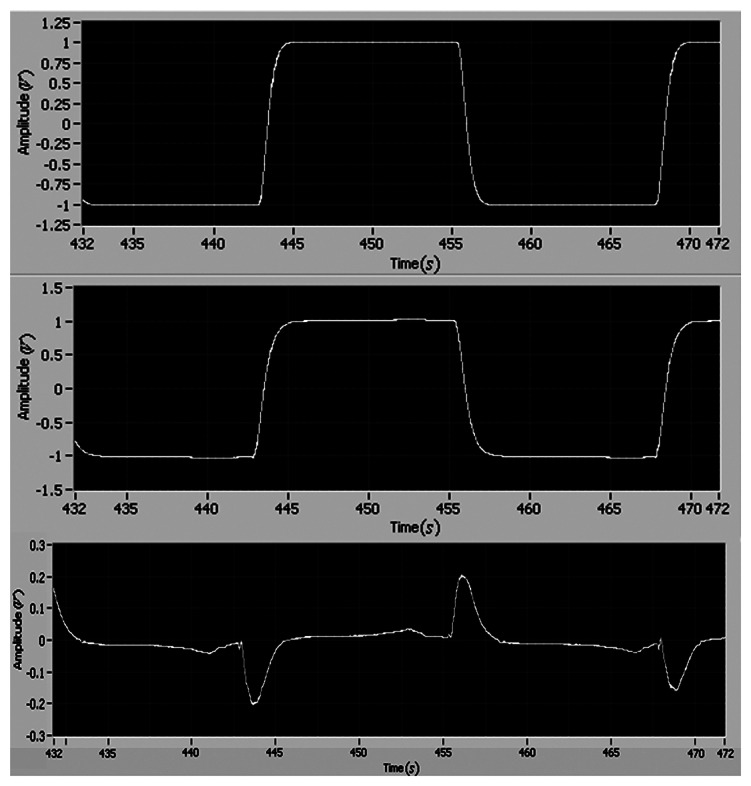
The reference position, the motor position and the tracking error (*i.e.*, (**top**), (**middle**) and (**bottom**), respectively).

**Figure 11.**
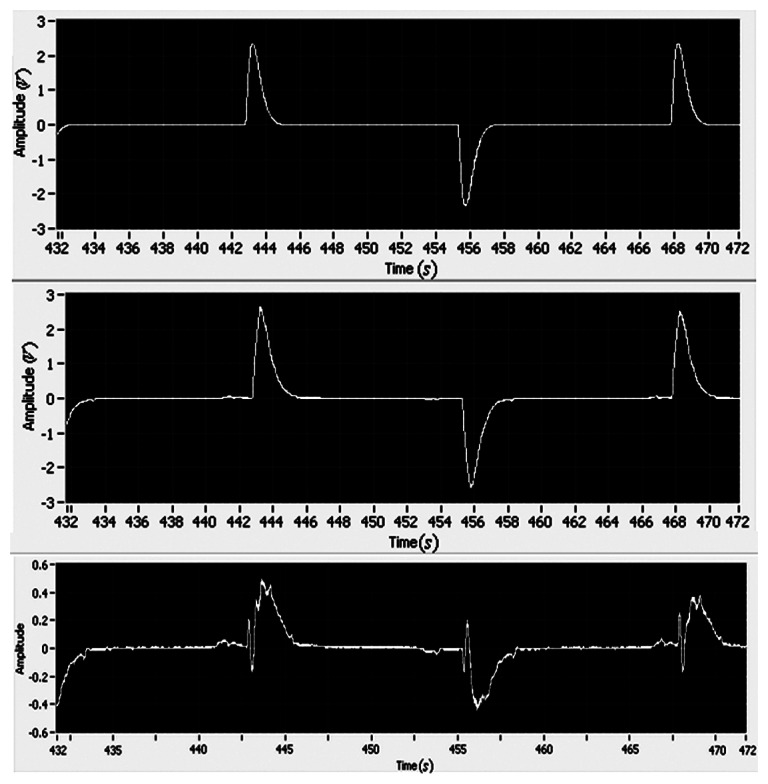
The reference velocity, the motor velocity and the tracking error (*i.e.*, (**top**), (**middle**) and (**bottom**), respectively).

The experimental results of the position and velocity tracking are shown in Figures 10 and 11, respectively. The maximum tracking errors of position and rate are less than 0.2 V and 0.4 V, respectively.

**Figure 12.**
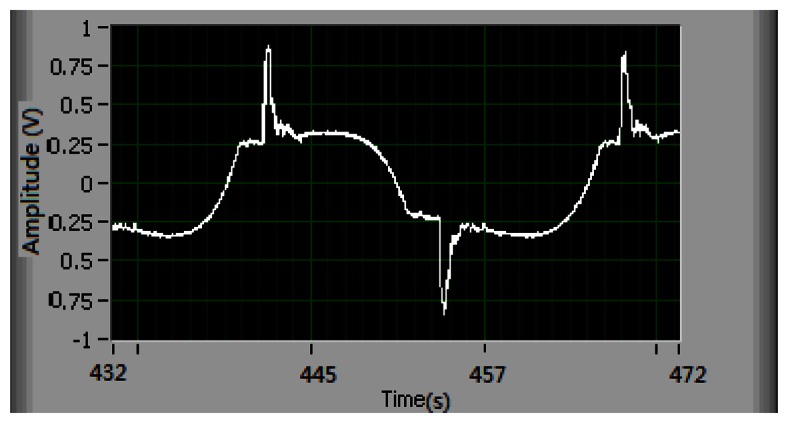
Control signal.

As shown in Figure 12, the magnitude of the controller is less than 0.85 V, and there is no chatter phenomenon. Compared to the former adaptive control without the integral action, the case with the integral adaptive control can reduce the reference errors of position and velocity by as much as 50%. The experimental results demonstrate that the integral action is effective to improve the tracking performance.

### 3.4. Tracking Performance with Input Disturbance

**Figure 13.**
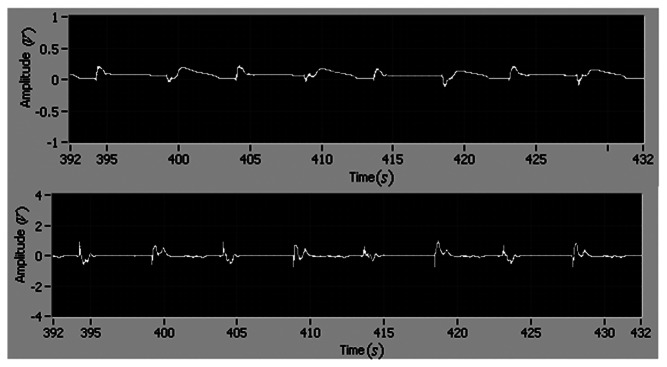
Tracking errors of position (**top**) and velocity (**bottom**).

In order to investigate the performance of the adaptive control, the input disturbance is considered in this section. The square wave disturbance with magnitude 1 is adopted. The reference is also square wave. During the experiment, the disturbance is added by using a LabView block as an input disturbance. The disturbance enters the close loop systems as the reference signal enters. The tracking errors of position and velocity are shown in Figure 13. It is seen that the maximum position and velocity tracking errors are less than 0.2 and 1, respectively. Also, it can be found that there is no difference between the two results of the position tracking errors shown in Figures 10 and 13, respectively. The position error is the most important one for the motor tracking in this paper, thus it can be concluded that the degradation of tracking performance is not significant. The experimental result indicates that the adaptive control with the integral action suppresses disturbance and tracks the reference simultaneously.

### 3.5. Comparison between Experiment and Simulation Results

**Figure 14.**
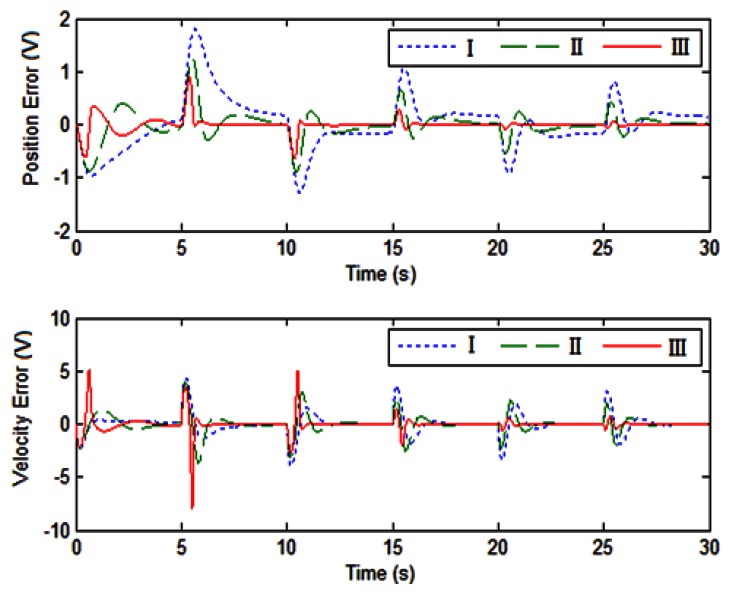
Comparison of experiment and simulation results (case I: experimental results of Adaptive control; case II: experimental results of adaptive control with integral action; and case III: Simulation results of simulation of adaptive control with integral action).

Finally, a comparison between experiment and simulation results is presented in this paper. According to the motor manual, the time constant is 0.25. The DC gain is 5.2. Figure 14 shows the comparison between experiment and simulation results.

The simulation and experiment have similar responses. The tracking errors of the position and velocity from experiment study are larger than that from simulation study. In the three cases, all the tracking errors asymptotically converge (*i.e.*, the stability can hold). The simulation of the proposed adaptive control with the integral action gives the best performance. The tracking error from simulation study can converge to zero, but the tracking errors from experiment study cannot converge to zero due to the friction, the sensor noise and the motor dead-zone. In the experiment study, the performance degradation is acceptable.

## 4. Conclusions

In order to accurately control motors, this paper employs both a rotary encoder and tachometer sensors to measure the angle-position and speed, respectively. Based on the measurements, two adaptive controllers are developed for the motor system. The stability and convergence are validated by the Lyapnov theorem and Barbalat lemma. Then, the control system is implemented by using Labview. Experimental results indicate that the tracking errors of the motor position and velocity asymptotically converge to their error ranges. In the presence of disturbances, the adaptive controller with integral action presents better performance than the case without integral action.

State estimation approaches are encouraged to investigate for possible output feedback control. In this paper, the proposed adaptive controllers needs full state information, but actually some states (e.g., motor velocity) are not provided. Thus, the state estimation must be designed in the possible output feedback control.

## References

[1] Khorrami F., Ksishnamurthy P., Melkote H. (2003). Modeling and Adaptive Nonlinear Control of Electric Motors.

[2] Casey N.F., Laura P.A.A. (1997). A review of the acoustic emission monitoring of wire rope. Ocean Eng..

[3] Casey N.F., Taylor J.L., Holford K.M. (1985). Wire break detection during tensile fatigue testing of 40 mm wire rope. Br. J. Non-Destr. Test..

[4] Casey N.F., White H., Taylor J.L. (1985). Frequency analysis of the signals generated by the failure of constituent wires of a wire rope. NDT Int..

[5] Hu D.J., Burg T. (1998). Nonlinear Control of Electric Machinery.

[6] Wiberg J. (2003). Controlling a Brushless DC Motor in a Shift by Wire System. MS.c Thesis.

[7] Nikulin G.L., Frantsuzova G.A. (2008). Synthesis of an electric-power steering control system, optoelectronics. Instrum. Data Process..

[8] Chaiya U., Kaitwanidvilai S. Fixed-Structure Robust DC Motor Speed Control.

[9] Xu D.G., Yang G. (2004). A simple and robust speed control scheme of permanent magnet synchronous motor. J. Control. Theory A.

[10] Nouri K., Dhaouadi R., Braiek N. (2008). Adaptive control of a nonlinear DC motor drive using recurrent neural networks. Appl. Soft Comput..

[11] Melkote H., Khorrami F. (1999). Nonlinear adaptive control of direct drive brushless DC motors and applications to robotic manipulators. IEEE Trans. Mechatron..

[12] Chen C.W. (2011). A fuzzy AHP-based fault diagnosis for semiconductor lithography process. Int. J. Innov. Comput. I.

[13] Chen C.W. (2011). Stability analysis and robustness design of nonlinear systems: An NN-based approach. Appl. Soft. Comput..

[14] Chen C.W. (2011). Stabilization of adaptive neural network controllers for nonlinear structural systems using a singular perturbation approach. J. Vib. Control.

[15] Chen C.W. (2010). GA-based adaptive neural network controllers for nonlinear systems. Int. J. Innov. Comput. I.

[16] Fallahi M., Azadi S. Adaptive Control of a DC Motor Using Neural Network Sliding Mode Control.

[17] Xu J.-X., Huang D., Venkataramanan V., Tuong H.T.C. Extreme Precise Motion Tracking of Piezoelectric Positioning Stage Using Sampled-Data Iterative Learning Control.

[18] Astrom K.J., Wittenmark B. (1994). Adaptive Control.

[19] Dursun M., Ozden S. Design of Monitoring System for Linear Switched Reluctance Motor with Quadrature Encoder and Current Sensors.

[20] Khail H.K. (2002). Nonlinear Systems.

